# The Androgen Dehydroepiandrosterone Sulfate Shows a Greater Relationship with Impulsivity than Testosterone in a Healthy Male Sample

**DOI:** 10.3390/brainsci14060569

**Published:** 2024-06-03

**Authors:** Anton Aluja, Ferran Balada, Óscar García, Neus Aymamí, Luis F. García

**Affiliations:** 1 Department of Psychology, University of Lleida, 25001 Lleida, Spain; 2 Lleida Institute for Biomedical Research, Dr. Pifarré Foundation, 25198 Lleida, Spain; ferran.balada@uab.cat (F.B.); oscar.garcia@universidadeuropea.es (Ó.G.); naymami@gss.cat (N.A.); luis.garcia@uam.es (L.F.G.); 3 Department of Biological and Health Psychology, Autonomous University of Barcelona, 08193 Bellaterra, Spain; 4 Department of Psychology, European University of Madrid, 28670 Madrid, Spain; 5 Psychiatry, Mental Health and Addictions Service, Santa Maria Hospital of Lleida, 25198 Lleida, Spain; 6 Department Psychobiology and Methodology of the Health Science, Autonomous University of Madrid, 28049 Madrid, Spain

**Keywords:** impulsivity personality trait, dehydroepiandrosterone sulfate (DHEA-S), testosterone, BIS-11, UPPS-P

## Abstract

This study was designed to examine the relationships among the impulsivity construct as a personality trait, the dehydroepiandrosterone sulfate (DHEA-S), and testosterone in a sample of 120 healthy middle-aged males (M_age_ = 44.39; *SD* = 12.88). The sum of the three BIS-11 scales, the SR, and the five UPPS-P scales correlated with DHEA-S 0.23 (*p* < 0.006) and testosterone 0.19 (*p* < 0.04), controlling for age. Partial correlations showed that DHEA-S was significantly related to motor impulsivity (0.24; *p* < 0.008), Sensitivity to Reward (0.29; *p* < 0.002), Lack of Premeditation (0.26; *p* < 0.05), and, to a lesser extent, Sensation Seeking (0.19; *p* < 0.04) and Positive Urgency (0.19; *p* < 0.04). Testosterone correlated with attention impulsivity (0.18; *p* < 0.04), Sensation Seeking (0.18; *p* < 0.04), and Positive Urgency (0.22; *p* < 0.01). Sensitivity to Reward, Negative Urgency, and Positive Urgency were significant predictors of DHEA-S (R^2^ = 0.28), and Positive Urgency for testosterone (R^2^ = 0.09). Non-parametric LOESS graphical analyses for local regression allowed us to visualize the non-linear relationships between the impulsivity scales with the two androgens, including non-significant trends. We discuss the implications of these results for impulsive biological personality traits, the limitations of our analyses, and the possible development of future research.

## 1. Introduction

In psychology research, the impulsivity trait has been studied in different personality models and personality theories that relate it to different behavioral dispositions such as precipitation, lack of anticipation, or Sensation Seeking [1,2,3]. Various models have set out to describe the components of impulsivity. Barrat [4] proposed a three-factor impulsivity model, and Dickman [5] suggested differentiating between functional and dysfunctional impulsivity. Human personality structural models also present different views about the impulsivity construct. For instance, Eysenck located impulsivity in the Psychoticism super trait [6,7], but later, Gray, extending Eysenck’s theory, located it in the high-Neuroticism and high-Extraversion quadrant, describing impulsivity as a component of Sensitivity to Reward, according to the Reinforcement Sensitivity Theory (RST) [8,9]. In the Five Factor Model, impulsivity is mainly considered the inverse pole of the Conscientiousness trait, but Neuroticism presents a facet named Impulsiveness in the NEO-PI-R [10]. This is a good example of the different nature of the various components of impulsivity. Considering the varying approaches to the concept of impulsivity, it could be concluded that this construct is not unidimensional and involves various sub-traits with moderate relationships among them [11,12].

Impulsivity is an important psychological correlate of risk behaviors [13,14,15]. It is well established that impulsivity and aggression are linked. Along this line, a meta-analysis showed significant correlations between facets of the UPPS-P Impulsive Behavior Scale and several different forms of aggression [16], and cognitive and motor impulsivity were predictors of self-reported total aggression [17]. Given the relevance of this construct to predict and explain several relevant outcomes, it is not surprising that some specific (mono-trait) measures have been developed. Self-report measures addressed to exclusively measure impulsivity are the UPPS-P Behavior Scale (this instrument also includes a scale of Sensation Seeking) [11], or the Barratt Impulsivity Scale, BIS-11 [18]. From instruments developed after the structural human personality models, a scale addressed to measure impulsivity is the Reward Sensitivity Scale (SR), from the Sensitivity to Punishment and Sensitivity to Reward Questionnaire (SPSRQ) [19,20]. The SR is related to Eysenck’s Psychoticism and impulsivity and Zuckerman’s Sensation Seeking scales [19,20,21,22]. Gray’s BAS is a neurobehavioral system that depends on dopamine-supplied structures and mediates individual differences in sensitivity and reactivity to appetitive stimuli associated with the BAS and impulsivity [23]. It should be noted that dopamine activity increases impulsivity [24]. In Zuckerman’s personality model, impulsivity was a facet of the broader Impulsive Sensation Seeking trait (ImpSS) [3].

Impulsive personality traits are heritable (40–60%) [25,26] and are related to the frontal–subcortical circuitry. In this way, subscales of both the UPPS-P and BIS-11 showed strong genetic correlations with phenotypic behaviors characterized by high impulsivity, such as drug addictions and attention deficit hyperactivity disorder (ADHD) [27]. At the molecular genetic level of analysis, it has been shown, for instance, that motor and non-planning impulsivity scales in BIS-11 were associated with two single nucleotide polymorphisms (SNPs) within the 5-HT2a receptor gene [28]. The androgen receptor (AR) gene has been linked to disinhibited impulsive personalities in male prison inmates measured through a combination of the following personality scales: Sensation Seeking, Aggression–Hostility, Psychoticism, Sensitivity to Reward, Novelty Seeking, and impulsivity. Inmates carrying CAG-short and GGN-long (trinucleotide repeat polymorphisms) haplotype groups (short–long haplotypes) obtained significantly higher scores on the impulsive–disinhibited index [21]. The interaction between free testosterone and CAG, and between sex-hormone-binding globulin testosterone transporter (SHBG) and CAG explained some of the differences in impulsivity. This occurred mostly in the group of short CAG repetitions and motor impulsivity [29]. Human aggression/impulsivity-related traits have a complex background that is greatly influenced by genetic and non-genetic factors [30].

Dehydroepiandrosterone sulfate (DHEA-S) is an anabolic steroid secreted by the adrenal cortex and is a precursor of testosterone and estrogens [31]. DHEA-S is produced in the zona reticularis of the adrenal cortex by the action of adrenocorticotrophic hormones (ACTHs). DHEA-S levels peak in young adulthood, and then decline progressively by 2–4% per year [32]. DHEA-S has been associated with different personality questionnaires related to impulsivity. Do Vale et al. [33] studied the relationship between DHEA-S and the combination of the psychopathic deviance and hypomania scales of the Minnesota Multiphasic Personality Inventory (MMPI) [34]. These two scales are considered to be indicators of impulsivity [35]. Presence of Borderline Personality Disorder (BPD), a disorder with predominant impulsivity, is also associated with high concentrations of DHEA-S in relation to subjects without personality disorders [36]. In a study with attention-deficit/hyperactivity disorder (ADHD) patients and controls, salivary DHEA levels were related to distractibility and impulsivity scores on the Continuous Performance Test (CPT). The authors concluded that DHEA-S might be a biomarker for ADHD [37]. In another study, morning DHEA-S levels were significantly higher in borderline subjects than controls [38]. In this sense, DHEA-S has been pointed to as a biomarker of acute stress [39], and it was significantly and positively associated with anger [40].

Testosterone production is primarily dependent on luteinizing hormones (LH) acting on the conversion of cholesterol to pregnenolone within the mitochondria of Leydig cells [41]. Testosterone levels also decline with age, while LH levels rise slightly or remain unchanged. The decline in testosterone with age is associated with a drop in energy level, muscular strength, physical, sexual, and cognitive functions, and mood [42]. In men, the percentages of testosterone decrease 1% per year from the age of forty [43]; 4% of testosterone is converted to dihydrotestosterone via a reductase enzyme and 0.2% to estradiol via the aromatase enzyme [44]. In women with polycystic ovarian syndrome, significant relationships were found between total testosterone (TT) levels and motor impulsivity and non-planning impulsivity [40].

Significant relationships between impulsivity and Sensation Seeking and testosterone have been reported in general and in criminal samples [45,46]. Thus, it has been replicated that subject with high scores on impulsive-related traits such as Experience Seeking, Disinhibition, or Boredom Susceptibility tended to present higher testosterone scores [21,29,47,48,49,50]. These studies support the theoretical association between Impulsive Sensation Seeking and gonadal hormones raised by Zuckerman’s psychobiological personality model [51]. Recently, exogenous testosterone supplementation has been found to be associated with trait impulsivity [52,53,54].

Despite the evidence relating both testosterone and DHEA-S with impulsivity and related personality characteristics, few studies have examined the relationship between impulsivity and testosterone and DHEA-S altogether. Moreover, since testosterone and DHEA-S androgens are related, it is necessary to simultaneously explore the role of both androgens in the differences observed in impulsivity. Thus, the main objective of this study was to examine the relationships between DHEA-S and testosterone and impulsivity simultaneously in a sample of healthy middle-aged men. Based on the studies reviewed, a moderate relationship was expected among both androgens (DHEA-S and testosterone) and impulsivity scales.

## 2. Method

### 2.1. Participants and Procedure

The participants in this study were 120 voluntary healthy men (M_age_ = 44.39; *SD* = 12.88), who received EUR 25 for their participation. They were part of the teaching and service administration staff of the university and were invited to participate through a collective email. Participants filled out the online personality questionnaires and provided two saliva samples for hormonal analysis (see below). All subjects were interviewed by a clinical psychologist who asked about possible medical or psychiatric history. Only healthy subjects who were not taking psychotropic or hormonal medications were admitted. The participants received oral and written information on the characteristics of the research before they signed a written consent form. The study was part of a national project and was authorized by the university’s ethics committee and data protection commission.

### 2.2. Impulsive Personality Traits Measures

#### 2.2.1. The Barratt Impulsiveness Scale (BIS-11) Is a 30-Item Questionnaire Comprising Three Scales: Attention (AI), Motor (MI), and Non-Planning (NPI) Impulsiveness [18]

The answer format was a 4-point scale ranging from 1 to 4. In a Spanish validation study, it was reported that the average Cronbach’s alpha reliability coefficient of the BIS-11 was 0.88. The factorial structure of the three factors was confirmed, and adequate convergent validity was obtained [55]. The authors concluded that the instrument was valid for research in the Spanish cultural context.

#### 2.2.2. The Impulsive Behavior Scale (UPPS-P) Shortened Version Was Originally Developed by Whiteside and Lynam [11]

Different versions of the UPPS instrument have been developed. The short version used in this research has 20 items and five scales: Negative Urgency (NU), Lack of Premeditation (PR), Lack of Perseverance (PS), Sensation Seeking (SS), and Positive Urgency (PU). It has robust psychometric properties with high internal consistency across different languages and cultures [56]. The Spanish version was used in the present study. A confirmatory factor analysis replicated the five-factor model of the original scale. The internal consistency of the scales ranged between 0.61 and 0.81. The scale has a 4-point Likert-type response format: 1 = strongly agree to 4 = strongly disagree [57].

#### 2.2.3. The Short Version of Sensitivity to Reward Questionnaire (SR) Is Part of the Sensitivity to Reward and Sensitivity to Punishment, Shortened 20-Item Version (SPSRQ-20) [20]

The answer format ranges from 1 to 4 points. The long 48-item questionnaire was developed by Torrubia et al. [19]. The SPSRQ-20 retains Sensitivity to Reward as a measure of impulsivity and the Behavioral Approach System (BAS) according to Gray’s theory. The short 10-item SR scale has an alpha consistency of 0.73, a value similar to that reported for the long version (24-item) in men (0.80).

### 2.3. Hormone Assays

The subjects went to the laboratory and received written instructions on how to collect the saliva samples at home over the following days between 8 and 9 o’clock in the morning. The saliva sample was obtained 30 min after getting up without having ingested food, liquids, or brushing teeth in two different tubes (one for DHEA-S and the other for testosterone). They were given a portable cooler to transport the refrigerated saliva sample from their home to the laboratory located on the university campus. The saliva sample for DHEA-S was collected via a cotton Salivette Sarstedt kit. The samples collected by a Sali-tube 100 (SLV-4158) were frozen and stored in the laboratory at −86 °C until the subsequent analysis using an ELISA technique (Salimetrics, State College, PA, USA), with each sample being analyzed in duplicate. The normal DHEA-S range level was 2.0–10.0 ng/mL, and the testosterone range was between 6.1 and 230.9 pg/mL. For DHEA-S, the inter-assay coefficient of variation (CV) was 9.66%, and 5.09% for testosterone, respectively.

### 2.4. Data Analysis Strategy

The sample was distributed into three groups based on age using the 33.3 and 66.6 percentiles as cut-off criteria (<37, 38–50, and over 50 years old). Testosterone and DHEA were log-transformed to base 10 due to their non-normal distribution and skewness and kurtosis values. A One-Way ANOVA and Scheffé Post Hoc Test were performed to compare the group means for the variables studied. Kurtosis, skewness, and Cronbach’s alpha values were also calculated. Frequency distribution values can be used as a test of normality. Normality was rejected if kurtosis and skewness exceeded the range of ±2 [58,59,60].

The relationships between the hormonal and psychometric variables were analyzed using an empirical network analysis (GLASSO, EBIC, and mgm algorithm) [53,61,62]. This technique makes it possible to estimate the partial correlations between each pair of domains while controlling for Type I error inflation and the presence of spurious correlations [63,64,65]. A factor component analysis with an orthogonal rotation of two factors was also carried out to verify the relationships between the impulsivity, age, and sex hormone scales.

The predictive power of each psychometric variable (impulsivity scales), including age, was computed separately on DHEA-S and testosterone using a multiple linear regression model. The enter method was performed with the usual PIN criterion (probability of F to enter; *p* < 0.05) and POUT (probability of removing F; *p* < 0.10). Lastly, to detect non-linear patterns, a non-parametric local LOESS graphic analysis was performed [66]. This polynomial regression procedure allows the production of data points for the DHEA-S and testosterone hormones (T-scores) based on the psychometric variables (Z-scores) to continuously observe the progression of the impulsivity variables as improvements are made in the hormone score. This implies a series of local regressions that allows a curved shape to vary across a continuous variable. The procedure is a robust and flexible fitting method and is ideal for observing trends or tendencies and revealing potentially complex and unexpected patterns of association between variables [67].

## 3. Results

### 3.1. Age Group Comparison, Frequencies, Distribution Values, and Internal Consistency

Table 1 shows the descriptive and mean comparisons of the hormones and impulsivity scales in the three age groups of the sample and the statistical significance for each group on the Scheffé test. The group of youngest subjects showed a higher mean in DHEA-S (*p* < 0.001) than the middle group, and the middle group a higher mean than the oldest (*p* < 0.007). In contrast, testosterone was higher in the younger group compared to that in the older group (*p* < 0.01). Regarding the impulsivity variables, the SR showed higher scores in the younger group compared to that in the older one (*p* < 0.01), and the middle group compared to that in the older one (*p* < 0.05). Young people were more likely to be sensation seekers than older ones (*p* < 0.002). For the other impulsivity variables, no statistically significant differences were observed, but there was a tendency for the youngest to be more impulsive. Kurtosis gave a range between −0.99 and 0.79, except for testosterone, which had a value of 1.8. Skewness gave a range between −0.05 and 0.94, and alpha internal consistency between 0.62 and 0.85.

### 3.2. Partial Empirical Network Analysis

**Figure 1:**
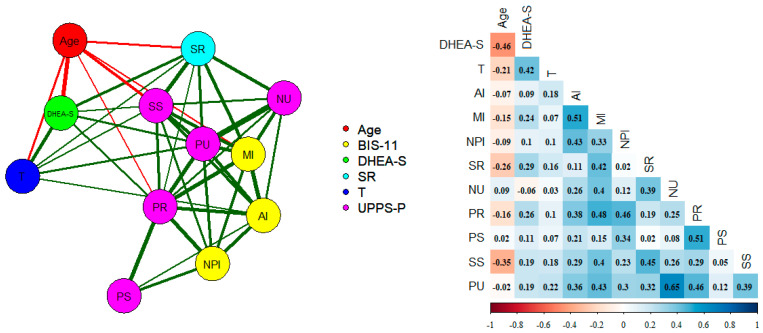
Empirical network with the age, testosterone, DHEA-S, UPPS-P, and SR domains (partial correlations). Nodes represent domains. The edges represent the relationship among the domains. The thicker the edge, the greater the relationship between the domains. Green and red lines represent positive and negative relationships, respectively. AI: Attention, MI: motor (MI), NPI: Non-Planning, SR: Sensitivity to Reward, NU: Negative Urgency, PR: Lack of Premeditation, PS: Lack of Perseverance, SS: Sensation Seeking (SS), and PU: Positive Urgency.

Figure 1 shows a graph with the partial correlations between the hormonal and psychometric variables included in the study and the statistical significance. As expected, DHEA-S and testosterone correlated positively (0.42; *p* < 0.001) and both correlated negatively with age (−0.46 and −0.21; *p* < 0.001). Sensation Seeking was negatively correlated with age (−0.35; *p* < 0.001). DHEA-S was significantly related to motor (0.24; *p* < 0.008), Sensitivity to Reward (0.29; *p* < 0.002) and Lack of Premeditation (0.26; *p* < 0.05), and to a lesser extent Sensation Seeking and Positive Urgency (0.19; *p* < 0.04). Testosterone correlated with SR (*p* < 0.04), Sensation Seeking (0.18; *p* < 0.04), and Positive Urgency (0.22; *p* < 0.01). The sum of the three BIS-11 scales, the SR, and the five UPPS-P scales correlated, 0.23 (*p* < 0.006) and 0.19 (*p* < 0.04), with DHEA-S and testosterone, respectively, controlling for age.

### 3.3. Principal Component Analysis

Two principal component analyses (PCAs) were performed with variables including hormonal variables and impulsivity variables retaining two factors. Age was also included in the first analysis, but not in the second. In the first PCA, factor I was integrated by seven impulsivity scales from both questionnaires, and factor II by DHEA-S, age, testosterone, Sensitivity to Reward, and Sensation Seeking. These last two scales had lower secondary loadings, but high ones in factor I, and motor impulsivity had a loading of 0.31 in factor II. In the second PCA, excluding age, factor II also included Sensitivity to Reward and Sensation Seeking together with DHEA-S and testosterone, but the scales of the Positive and Negative Urgencies and motor impulsivity were also integrated (Table 2).

### 3.4. Impulsivity and Age as a Hormonal Prediction Power

Table 3 shows a multiple linear regression analysis taking the three BIS-11 scales, the five UPPS-P scales, and age as independent variables, and DHEA-S and testosterone as dependent variables using the enter method. Standardized coefficients for DHEA-S showed a significant beta for age (*p* < 0.001), Sensitivity to Reward (*p* < 0.037), Negative Urgency (*p* < 0.003), and Positive Urgency (*p* < 0.008) with a final adjusted R^2^ = 0.28. The most predictive variables for testosterone were age (*p* < 0.027) and Positive Urgency (*p* < 0.007) with a final adjusted R^2^ = 0.09.

### 3.5. Non-Parametric Local LOESS Graphic Analysis

**Figure 2:**
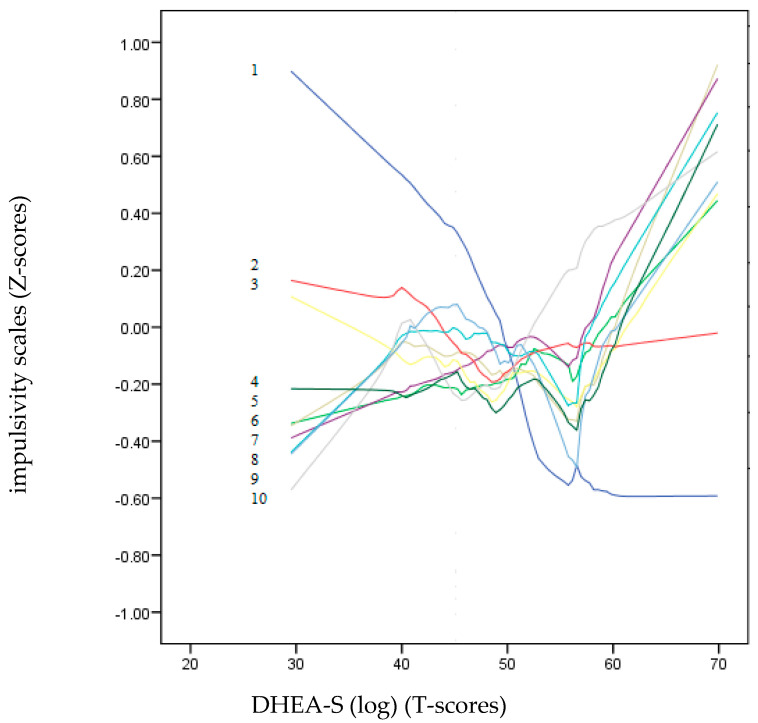
LOESS plots for DHEA-S (T-score) (dependent variable) and impulsivity scales (independent variables): 1: Ae, 2: Lack of Premeditation, 3: Attention, 4: Non-Planning, 5: Positive Urgency, 6: Motor, 7: Negative Urgency, 8: Sensation Seeking, 9: Lack of Perseverance and 10: Sensitivity to Reward (*Z*-score). The positive predictions of the independent variables on the dependent variable (DHEA-S) are located on the lines above “zero” T-scores.

**Figure 3:**
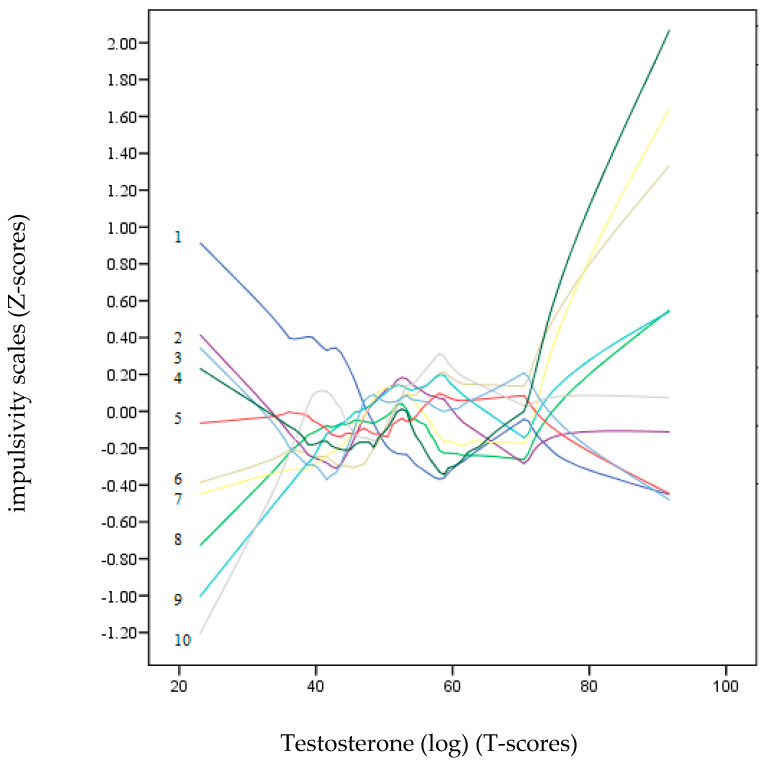
LOESS plots for testosterone (T-score) (dependent variable) and impulsivity scales (independent variables): 1: Age, 2: Lack of Premeditation, 3: Lack of Perseverance, 4: Non-Planning, 5: Negative Urgency, 6: Positive Urgency, 7: Attention, 8: Motor, 9: Sensation Seeking, and 10: Sensitivity to Reward (*Z*-score). The positive predictions of the independent variables on the dependent variable (testosterone) are located on the lines above “zero” T-scores.

Figure 2 and Figure 3 show a non-parametric LOESS graphical analysis for local regression. The BIS-11 and UPPS-P scales are represented in Z scores, whereas the scores of the hormones (DHEA-S and testosterone) are on a T-score scale. These graphs allowed us to observe the non-linear progress of the impulsivity scales (positive and negative) as the hormone levels increased. These curves indicated a variety of non-linear trends for most of the impulsivity scales. In Figure 3, the age dropped drastically as the value of DHEA-S increased. Except for Lack of Perseveration, which remained around the zero value of the Z-score axis, all the other impulsivity scales showed a strong upward trend towards positive points as the DHEA-S value increased. In contrast, in Figure 3, most of the impulsivity scales remained at zero or at slightly negative Z-scores, except for motor, Attention, and Positive Urgency, which tended to be placed in positive Z-score positions.

## 4. Discussion

The main goal of this study was to examine the relationship between two androgenic steroids, DHEA-S and testosterone, and the impulsivity trait measured with different instruments. Preliminary results showed a significant correlation between the two androgens, as expected, and a significant negative correlation with age [32,43]. Hormone means and ranges fit the values expected in a normal population. High skewness was as expected, so the values were logarithmically transformed as is customary in a hormone study. Logarithmic transformations tend to normalize the distribution of hormones considering the distances between the different values [68]. Regarding psychometric measures of impulsivity, it was also observed that the BIS-11 and the UPPS-P scales did not represent a one-dimensional construct [11,12]. Along this line, for example, non-significant correlations were obtained between impulsivity scales such as Negative Urgency, motor impulsivity, Non-Planning, Lack of Perseverance, and Negative Urgency.

Age was negatively related to impulsive personality traits, with Sensation Seeking and Sensitivity to Reward being the variables with the highest partial correlations, controlling for the rest of the variables. Along this line, there were also significant associations between DHEA-S and motor impulsivity, Sensitivity to Reward, Lack of Premeditation, Sensation Seeking, and Positive Urgency. Testosterone correlated with Sensitivity to Reward, Sensation Seeking, and Positive Urgency. However, the sum of all the BIS-11, UPPS-P, and SR scales correlated significantly with DHEA-S and testosterone, demonstrating an association, albeit a weak one, between the broad construct of impulsivity with the two hormones. Several of the impulsivity scales were associated with the variance of DHEA-S (up to 28% of the variance) and, to a lesser extent (9%), with the variance of the testosterone. Therefore, a moderate relationship between the measures of impulsivity with the two androgens was confirmed. It should be remarked that DHEA-S presented a much stronger relationship with impulsivity scales than did testosterone.

Additionally, in this study, the non-linear relationships between the two hormones and the impulsivity scales were also examined using a non-parametric LOESS graphical regression. LOESS curve (local polynomial regression) is a method of fitting a smooth curve between two variables [69]. This method combines the simplicity of least-squares linear regression with the flexibility of non-linear regression. In reference to the relationships between the impulsivity variables and DHEA-S, the graph clearly shows that as the DHEA-S values increased, the impulsivity scales increased, except for that of Lack of Premeditation. On the other hand, in the testosterone graph, only Lack of Perseverance, Attention, and Positive Urgency showed a tendency.

As commented in the introductory section, the relationship in humans between aggressiveness and impulsivity with steroid hormones is moderate. However, biological theories of personality suggest that impulsivity interacts with traits such as Sensation Seeking or similar ones such as Cloninger’s Novelty Seeking [70,71,72,73]. Dopamine also plays a role in impulsive behavior and reward seeking, while serotonin plays an inhibiting role. Testosterone and dopamine are related; dopamine can influence testosterone, and testosterone can influence dopamine, and both of them play an important role in male sexuality. Crucial to health is male sexual function. One study found that the endogenous administration of dopamine agonists to the medial preoptic area of rats increased sexual activity [74]. Another study found that castrated male rats did not show sexual interest and did not release dopamine in the medial preoptic area. After testosterone injections, castrated rats had sexual intercourse and increased dopamine release in the medial preoptic area [75].

Following Zuckerman’s theory, it has been proposed that testosterone could have an antagonistic role in monoamine oxidase (MAO), allowing a higher concentration of activating catecholamine in receptors due to lack of degradation [76]. DHEA-S is also an inhibitor of MAO activity [77]. The BAS (impulsivity) is associated with the dopaminergic system, while the BIS (anxiety) is associated with the septo-hippocampal system and the amygdala. These structures have a high density of steroid receptors, so differences in personality can be expected [78]. The BAS system is a neurobehavioral system that depends on dopamine-supplied structures and mediates individual differences in sensitivity and reactivity to appetitive stimuli associated with the BAS and impulsivity [22]. Dopamine activity increases impulsivity [23]. Exogenous DHEA-S produces a significant increase in the levels of acetylcholine, norepinephrine, and dopamine in the brain [79].

Therefore, research on aggressivity/impulsivity and androgens may in the future provide new findings and explanations about their biological connection, including genetics, thanks to a potentially greater understanding of the functioning of the prefrontal lobe and the dopaminergic pathways of the brain. Studies with rats have suggested that the GABA A receptor may be associated with testosterone-mediated impulsivity [80]. In the current study, Sensitivity to Reward, and, to a lesser extent, Sensation Seeking, both of which having a considerable biological basis in the literature, were the variables most closely related to hormones.

This study had several limitations. It was a cross-sectional design, so no causal conclusions can be drawn. The sample size was moderate, and it is possible that these findings could be less significant in a larger sample, and as the sample was restricted to men, it precluded additional confounding factors such as biochemistry differences in androgens between males and females. In addition, other variables affecting androgen concentrations such as smoking, diet, alcohol consumption, physical activity, weight, height, and muscle were not controlled for, which could have affected the data and results. Finally, since the subjects volunteered for this study, it is possible that the results could not be generalized to the general population.

## 5. Conclusions

In conclusion, the present results support a moderate relationship between impulsivity and the androgenic steroids DHEA-S and testosterone, in line with the findings reported by investigators in male samples. These results were greatly affected by age, both in impulsivity levels and in androgen levels. Research on DHEA-S and impulsivity has been much scarcer than on testosterone. Our results reported a greater relationship between DHEA-S than testosterone with impulsivity. This consistent association of DHEA-S with impulsive or disinhibited personalities has been demonstrated by other researchers, who found that DHEA-S was directly related to the deviant behavior triad and type A personality [32] or borderline personality disorder subjects [37]. Considering the limitations outlined above, future studies should continue to study the role of DHEA-S in personality in general, and aggressive and impulsive behavior in particular. Variables such as dopamine, norepinephrine, cortisol/testosterone ratio, and cortisol/DHEA-s, GABA A receptor, androgen receptor (AR) genes should also be included.

## Figures and Tables

**Table 1:** Descriptive, ANOVA age group comparison, frequency distribution values, and internal consistency of scales.

	(1) *n* = 40		(2) *n* = 39		(3) *n* = 41					
	<38 Years		38 to 50 Years		<50 Years		*p*<			
	M	*SD*	M	*SD*	M	*SD*	Scheffé	K	S	α
Age	29.93	4.28	44.41	3.89	58.64	5.84	-	−0.99	0.11	-
DHEA-S *	0.86	0.20	0.76	0.24	0.61	0.18	1 > 2 (0.001); 2 > 3 (0.007)	−0.34	0.04	-
Testosterone *	2.09	0.17	2.01	0.21	1.97	0.16	1 > 3 (0.014)	1.8	0.44	-
Attention (BIS-11)	14.39	5.26	13.11	4.48	13.83	4.50		−0.08	0.56	0.62
Motor (BIS-11)	13.80	6.78	12.08	5.26	11.63	5.51		0.73	0.83	0.73
Non-planning (BIS-11)	15.63	7.01	15.24	5.97	14.76	7.48		0.79	0.94	0.72
Negative Urgency	7.80	1.91	7.47	2.33	8.12	3.12		0.11	0.49	0.80
Lack of Premeditation	8.10	2.08	7.74	2.10	7.44	2.21		−0.34	0.08	0.81
Lack of Perseverance	8.61	1.46	8.76	1.97	8.78	1.98		−0.41	0.07	0.69
Sensation Seeking	9.93	3.03	8.71	2.69	7.73	2.37	1 > 3 (0.002)	−0.60	0.07	0.85
Positive Urgency	7.46	2.42	6.97	2.68	7.07	2.59		0.27	0.77	0.83
Sensitivity to Reward	21.90	4.12	21.37	3.82	19.02	4.61	1 > 3 (0.010); 2 > 3 (0.05)	−0.68	−0.05	0.76

Note: * Log 10 transformed. M: Mean; *SD*: standard deviation; K; kurtosis; S: skewness; α: Cronbach’s alpha.

**Table 2:** Principal component analysis with varimax rotation with DHEA-S, testosterone, BIS-11, and UPPS-P, including and excluding age.

*Including Age*	I	II	*Excluding Age*	I	II
Positive Urgency	**0.72**	0.20	Non-Planning (BIS-11)	**0.76**	0.11
Lack of Premeditation	**0.72**	0.19	Lack of Premeditation	**0.76**	**0.32**
Motor (BIS-11)	**0.69**	**0.31**	Lack of Perseverance	**0.73**	−0.09
Attention (BIS-11)	**0.66**	0.10	Attention (BIS 11)	**0.59**	0.24
Negative Urgency	**0.65**	−0.01	Sensitivity to Reward	−0.12	**0.77**
Non-Planning (BIS-11)	**0.61**	0.03	Positive Urgency	0.30	**0.70**
Lack of Perseverance	**0.46**	−0.06	Sensation Seeking	0.13	**0.67**
DHEA-S	0.03	**0.77**	Negative Urgency	0.09	**0.66**
Age	0.09	**−0.76**	Motor (BIS11)	**0.35**	**0.64**
Testosterone	0.05	**0.57**	DHEA-S	0.12	**0.48**
Sensitivity to Reward	**0.31**	**0.55**	Testosterone	0.07	**0.42**
Sensation Seeking	**0.41**	**0.53**			

Note: Factor loadings values higher than 0.30 in boldface.

**Table 3:** Linear multiple regression analysis for gender including age, BIS-11, SR, and UPPS-P as independent variables, and DHEA-S and testosterone as dependent variables (standardized).

DHEA-S				Testosterone			
Adjusted R^2^ = 0.28	β	*t*	*p*<	Adjusted R^2^ = 0.09	β	*t*	*p*<
*(Constant)*		*4.92*	*0.001*	*(Constant)*		*14.14*	*0.001*
Age	−0.39	−4.39	**0.001**	Age	−0.22	−2.25	**0.027**
Attention (BIS-11)	−0.04	−0.40	0.690	Attention (BIS-11)	0.16	1.45	0.151
Motor (BIS-11)	0.15	1.34	0.183	Motor (BIS-11)	−0.11	−0.93	0.352
Non-Planning (BIS-11)	−0.05	−0.51	0.609	Non-Planning (BIS-11)	0	−0.02	0.982
Sensitivity to Reward	0.21	2.11	**0.037**	Sensitivity to Reward	0.15	1.30	0.196
Negative Urgency	−0.34	−3.05	**0.003**	Negative Urgency	−0.20	−1.59	0.115
Lack of Premeditation	0.05	0.46	0.649	Lack of Premeditation	−0.13	−1.04	0.300
Lack of Perseverance	0.09	0.91	0.365	Lack of Perseverance	0.07	0.66	0.510
Sensation Seeking	−0.13	−1.35	0.180	Sensation Seeking	−0.01	−0.09	0.927
Positive Urgency	0.32	2.71	**0.008**	Positive Urgency	0.36	2.73	**0.007**

Note: Significant *p*-values in boldface.

## Data Availability

The data presented in this study are available on request from the corresponding author as several unfinished doctoral theses were projected on this project and the dissemination of some of the data contained in the database may affect the authorship of these theses.
